# Differential determination of perceived stress in medical students and high-school graduates due to private and training-related stressors

**DOI:** 10.1371/journal.pone.0191831

**Published:** 2018-01-31

**Authors:** Rebecca Erschens, Anne Herrmann–Werner, Katharina Eva Keifenheim, Teresa Loda, Till Johannes Bugaj, Christoph Nikendei, Maria Lammerding–Köppel, Stephan Zipfel, Florian Junne

**Affiliations:** 1 Department of Psychosomatic Medicine and Psychotherapy, University Hospital Tuebingen, Tuebingen, Germany; 2 Centre for Psychosocial Medicine, Department of General Internal Medicine and Psychosomatics, University Hospital Heidelberg, Heidelberg, Germany; 3 Competence Center for University Teaching in Medicine, Faculty of Medicine, University of Tuebingen, Tuebingen, Germany; Robert Bosch Krankenhaus, GERMANY

## Abstract

**Objective:**

Numerous studies from diverse contexts have confirmed high stress levels and stress-associated health impairment in medical students. This study aimed to explore the differential association of perceived stress with private and training-related stressors in medical students according to their stage of medical education.

**Methods:**

Participants were high-school graduates who plan to study medicine and students in their first, third, sixth, or ninth semester of medical school or in practical medical training. The self-administered questionnaire included items addressing demographic information, the Perceived Stress Questionnaire, and items addressing potential private and training-related stressors.

**Results:**

Results confirmed a substantial burden of perceived stress in students at different stages of their medical education. In particular, 10–28% of students in their third or ninth semesters of medical school showed the highest values for perceived stress. Training-related stressors were most strongly associated with perceived stress, although specific stressors that determined perceived stress varied across different stages of students’ medical education. High-school graduates highly interested in pursuing medical education showed specific stressors similar to those of medical students in their third, sixth, or ninth semesters of medical school, as well as stress structures with heights of general stress rates similar to those of medical students at the beginning of practical medical training.

**Conclusions:**

High-school graduates offer new, interesting information about students’ fears and needs before they begin medical school. Medical students and high-school graduates need open, comprehensive information about possible stressors at the outset of and during medical education. Programmes geared toward improving resilience behaviour and teaching new, functional coping strategies are recommended.

## Introduction

Stress-associated mental health impairment in medical students has been an important issue in recent international research [1–4]. In general, psychological distress can be viewed as an unspecified bodily reaction to specific demands in specific or general situations [5]. According to Lazarus [6],“Stress arises when individuals perceive that they cannot adequately cope with the demands being made on them or with threats to their well-being”. Evidence shows that both medical students and physicians experience higher symptom loads of psychological distress, depression, anxiety, and professional burnout than the average population [1, 7, 8].

Several studies have suggested that stress varies over time and have reported different levels of distress in medical students at different stages of their medical education. Medical students experience stress from the beginning of their medical education [2, 9, 10], other studies reported that stress-levels of medical students peak in the middle stages of medical training [11–13] or increase steadily during medical school [11, 14, 15]. Of course, some degree of stress is normal and an unavoidable component of both medical education and working as a physician [1]. However, high, permanent levels of distress can eventually cause feelings of fear, incompetence, uselessness, anger, or guilt [16]. Moreover, permanently high stress levels can negatively affect cognitive processes [17], and chronic stress can impair attention [18] and reduce concentration. High levels of distress can also encourage alcohol and drug abuse [19–21], prompt conflicts in interpersonal relationships [12, 22, 23], and cause high symptom loads of depression, anxiety, burnout [1, 24–27], and physical burden [28, 29]. For medical students in particular, medical training-related distress also negatively affects academic performance [29, 30].

Despite mounting evidence of the prevalence of increased stress and associated disorders, evidence on specific stressors determining perceived stress in medical students remains limited, particularly regarding the differential role of private versus training-related stressors. By definition, *stressor* is a personal or environmental event that causes stress [31, 32]. Quantitative studies have found that sources of stress among medical students seem to relate to medical school in terms of academic performance and increased workload [12, 23, 33, 34]. However, private conditions including financial problems and separation from family are important stressors as well [14, 35, 36]. However, to date, no comprehensive direct comparison of the relevance of private and training-related stressors among medical students is available.

The presented study focuses on the direct comparison of private and training-related stressors that could cause distress in medical students at different stages of their medical education. The group of medical students was expanded to also include high-school graduates planning to study medicine. That cohort might reveal specific information about the height and characteristics of psychological distress in early stages of medical education. At the same time, no study has investigated the prevalence of psychological distress and specific stressors in high-school graduates planning to study medicine.

## Materials and methods

### Ethics statement

The Ethics Committees of the Faculty of Medicine at University Hospital Tuebingen approved the study (number 053/2014BO1). All medical students and high-school graduates provided their written informed consent.

### Design, participants, and procedure

The study was conducted at the University Hospital of Tuebingen using a cross-sectional design. A total of 1,425 participants were invited starting with the winter-semester (October) until the start of the summer-semester (April) from four groups: high-school graduates planning to study medicine; first-year medical students; medical students in their third, sixth, or ninth semester; and students during their final year. Students were recruited consecutively with equal distributions across the individual semester periods. Students preparing for state examinations (e.g., fourth and tenth semesters) were excluded to prevent inflated stress rates. According to this context, Nikendei and colleagues [37, 38] present an overview to the field of medical education in Germany. First-year medical students were recruited from an introductory course, whereas high-school graduates were recruited at the university’s on-campus recruitment day, attended by individuals interested in pursuing medical education. All students completed a paper version of the questionnaire made available. By slight contrast, students in final year medical training were recruited via an online version of the questionnaire, due to the expansive geographical distribution of clinics where students complete their practical training.

### Measurements

Dimensions of the questionnaire included demographic information: age, gender, stage of medical training, cohabitation status, and if students passed their A-level in Germany or in another country. The results section shows details regarding the response rates and demographics for each subgroup. Levenstein and colleagues’ [39] Perceived Stress Questionnaire (PSQ-20) was used to assess perceived stress as a dependent variable. Potential determinants of stress were assessed using a broad set of newly designed items based on qualitative material explained below and a systematic review of literature on the topic.

#### Perceived Stress Questionnaire (PSQ-20)

The PSQ-20 is a validated instrument for measuring general subjective experiences and perceived stress independent of a specific context [39]. In this study, the German short version of the PSQ-20 with 20 items was used [40]. Items on the PSQ-20 are rated on a 4-point Likert scale (1 = *Almost never*, 2 = *Sometimes*, 3 = *Often*, 4 = *Usually*). The PSQ-20 also entails four subscales: Worries, Tension, and Loss of Joy, which map the individual’s internal stress reactions, and Demands, which records the general perception of external stressors. PSQ-20 sum scores range from 0 to 80, and its internal consistencies in Cronbach’s alpha are .80–.86 [40].

#### Measurements of specific stressors

Items used to measure specific stressors were developed based on qualitative material consisting of the written reflections of medical students within a reflexive portfolio obtained from the Merlin Study [41] and results from focus groups and expert groups. The qualitative dimensions and resulting items were reflected on items from literature based on a review of literature on the topic, e.g. [1, 16, 42–44], and existing instruments, e.g. [45]. Specific stressors were grouped as either private or training-related stressors (Table 1). The final instrument included a total of 20 private- and training-related stressors that participants rated on a 5-point Likert scale (1 = *Causing no stress at all*, 5 = *Causing severe stress*) during the last 4 weeks.

**Table 1:** Investigated stressors of private-life, training-related stressors and demographic control factors in each subgroup.

	private-related-stressors	training-related-stressors
**all subgroups**	financial worries living situation conflicts in partnership Side job conflicts with friends sports/ hobbies childcare conflicts with parents	study-related time time management / working style missing consultation and support selection performance pressure professional requirements timetable
**high-school graduates**		timetable stress with A-level examinations contact with teachers contact with class mates
**freshman** **1**^**th**^ **semester**		timetable topics dealing with diseases, dying and death contact with academics contact with other medical students begin of medical training
**medical students** **(3**^**rd**^**, 6**^**th**^**, 9**^**th**^ **semester)**		timetable topics dealing with diseases, dying and death contact with lectures contact with other medical students
**students in final year**		hours to work topics dealing with diseases, dying and death contact with medical supervisors contact with other students in practical year contact with patients contact with nurses contact with colleagues

The investigated specific stressors were categorized into stressors related to private-life and training-related stressors. Furthermore, demographic control factors such as for example gender and migration status were included in order to control for their respective influence. The investigated stressors of private-life and control factors were held constant across all included subgroups. For the training-related stressors, a subset of factors was tailored respectively to the specific target group in question (for example for High School Students vs. Students in their Year of Practice at Medical School) (see Table 1).

#### Statistical analysis and model development

For descriptive summary statistics, means (*M*) and measures of standard deviations (*SD*) were calculated for PSQ-20 sum scores and the four subscales. If requirements for one-way analysis of variance were violated, then sample characteristics were analysed with the Kruskal–Wallis test, Mann–Whitney *U* test, and Pearson’s chi-square test. In accordance with reference population studies by Fliege and colleagues [40] and Kocalevent and colleagues [46], the study sample was divided into two groups—highly stressed (PSQ sum score >49) and low stressed (PSQ sum score ≤49)—for further analysis within regression models. Level of significance was set at *p* = .05 to indicate statistically relevant differences. To evaluate group differences of students in the high stressed group, we used chi-squared tests with Bonferroni correction for post hoc tests.

Individual means for single items of private and training-related stressors were aggregated into two sum scores: private-related stressors and training-related stressors (TRS). To investigate associations with perceived stress, private-life related stressors and training-related stressors, were correlated using Spearman rho procedures (r_s_) or Pearson product-moment correlation coefficient (r).

Stepwise calculations of regression models were applied to identify the most relevant specific stressors within the investigated set of factors at different stages of medical education. The PSQ-20 sum score served as the dependent variable, and single specific stressors were entered as independent model determinants. Students’ characteristics (e.g., age, gender, passed A-level in Germany or in another country, and cohabitation status) were included to control for demographic aspects. Adjusted R^2^ was used as the measure of total variation for the hypothesised model. The adjusted R^2^ is a modified version of R^2^ that has been adjusted for the number of predictors in the model. The adjusted R^2^ is corrected by the number of degrees of freedom and increases only if the new predictor improves the model more than would be expected by chance [47, 48].

Standardised *ß*-values are reported as measures of individual variance explained by single determinants (i.e., stressors). Statistical model assumptions of multivariate linear regression analyses were tested based on residual statistics and graphical analyses. To test for multicollinearity, we calculated detective tolerance and interpreted values >0.25 as acceptable, as well as the variance inflation factor (VIF) and interpreted values <4 as acceptable [49]. Possible autocorrelations among single stressors were tested using the Durbin–Watson test and values 1.5–2.5 interpreted as acceptable [50]. Graphic plots of residuals were checked for all included factors to monitor homoscedasticity and linearity. All statistical analyses were performed using the Statistical Package for the Social Sciences version 22 [51].

## Results

### Sample characteristics

Table 2 provides an overview of the characteristics of the study population A total of 1,092 (76.6%) of the 1,425 recruited students participated in the cross-sectional study, of whom 711 (65.1%) were women.

**Table 2:** Characteristics of the study population.

	high-school graduates	freshman	3^rd^ semester	6^th^ semester	9^th^ semester	students in final year	total/p-value
**RR**[answered/ invited]	[346:400]	[149:180]	[143:180]	[123:180]	[154:180]	[177:305]	[1,092:1,425]
%	86.5%	82.8%	79.4%	79.4%	85.6%	58.1%	76.6%
**gender**							
n (female:male)	254:92	85:64	90:53	67:56	98:56	117:60	p > .05
% (female:male)	73.4:26.6	57.0:43.0	62.9:37.1	54.5:45.5	63.6:36.4	66.1:33.9	p > .05
**age** (years)	17.0±1.81	22.13±3.13	22.28±3.57	23.05±4.00	26.42±3.92	27.77±4.10	p < .01**
[Min-Max]	[15–20]	[18–37]	[16–38]	[21–52]	[21–47]	[23–60]	p < .01**
**cohabitation status**							
n (single:in partnership)		78:71	73:70	68:55	102:52	116:58	p < .01**
% (single:in partnership)		52.3:47.7	51.0:49.0	55.3:44.7	66.2:33.8	65.5:34.5	p < .01**
**a-Level**							
n (others:Germany)		13:132	8:134	8:107	11:131	3:171	p < .01**
% (others:Germany)		7.4:92.6	5.6:94.4	6.5:93.5	7.2:92.8	1.7:98.3	

The response rate (RR) varied between 58.1% and 86.5% with a mean RR of 76.6%. As expected, the population consists of more female students and high school graduates. It is evident that there are fewer partnerships at higher levels of education. There are also fewer students in higher education who have completed their a-Level in a country outside Germany.

### Perceived stress at different stages of medical training

**Fig 1:**
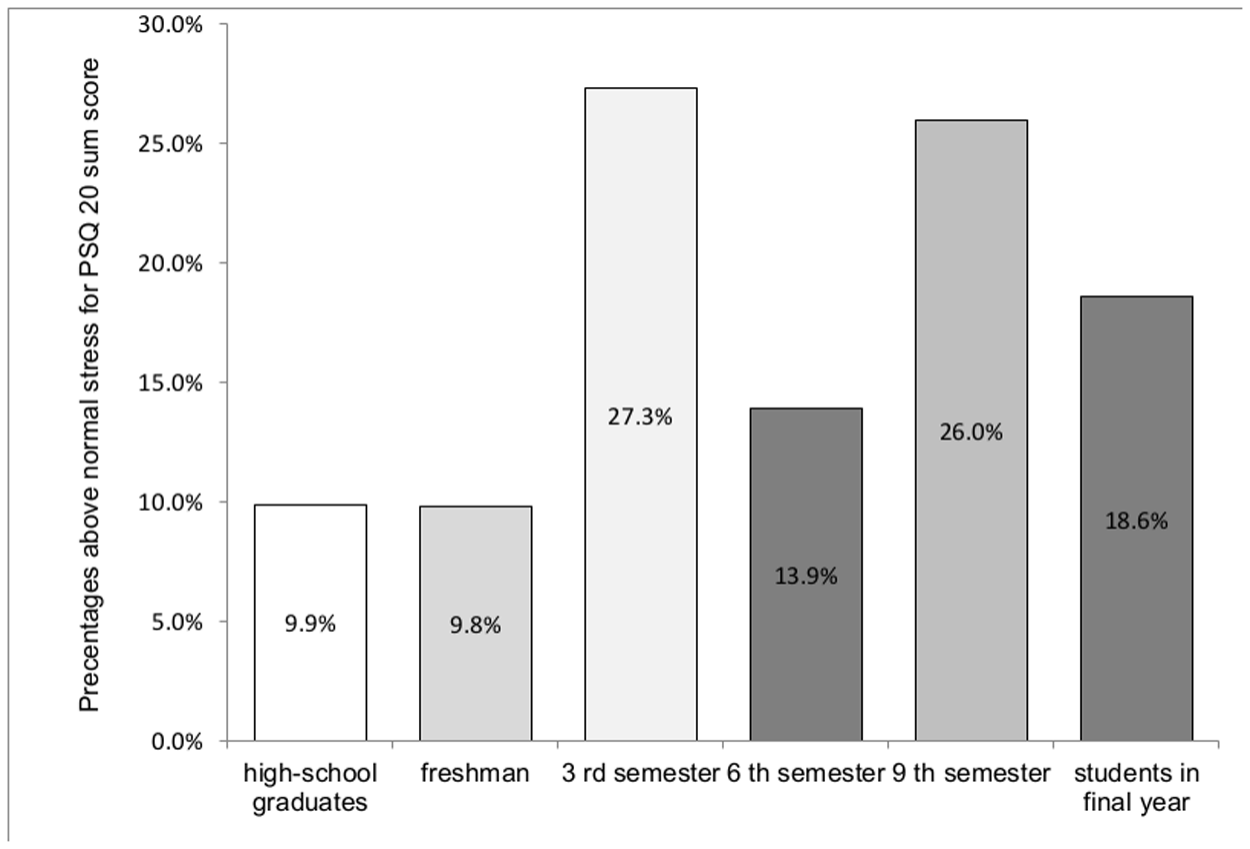
Illustration of the proportion of highly stressed students in different stages of medical training. Stress was measured with the PSQ-20. The bars represent the respective prevalence rate of the highly stressed students in 1st, 3rd, 6th and 9th semester, and students in practical year and high-school graduates with PSQ sum score >49. Significant differences emerged for levels of perceived stress among medical students. Post hoc analyses showed the following relative stress levels: 3^rd^ semester = 9^th^ semester > students in final year > 6^th^ > high-school graduates = freshman students.

Significant differences emerged for levels of perceived stress among students at those different stages (*Χ*^2^ = 39,7; *df* = 5; *p* < .01**). Post hoc analyses yield that students in 3^rd^ semester and 9^th^ semester achieved the highest proportion above the reference values of PSQ sumscore over 49 with prevalence rates between 26.0 and 27.3%. Students in final year had lower rates as these groups with 18.6%, but higher prevalence rates than high-school graduates (9.9%) and freshman students with 9.8%, which formed the lowest proportions (Fig 1).

#### Specific stress: Perceived demands, worries, tension, and loss of joy at different stages of medical education

**Fig 2:**
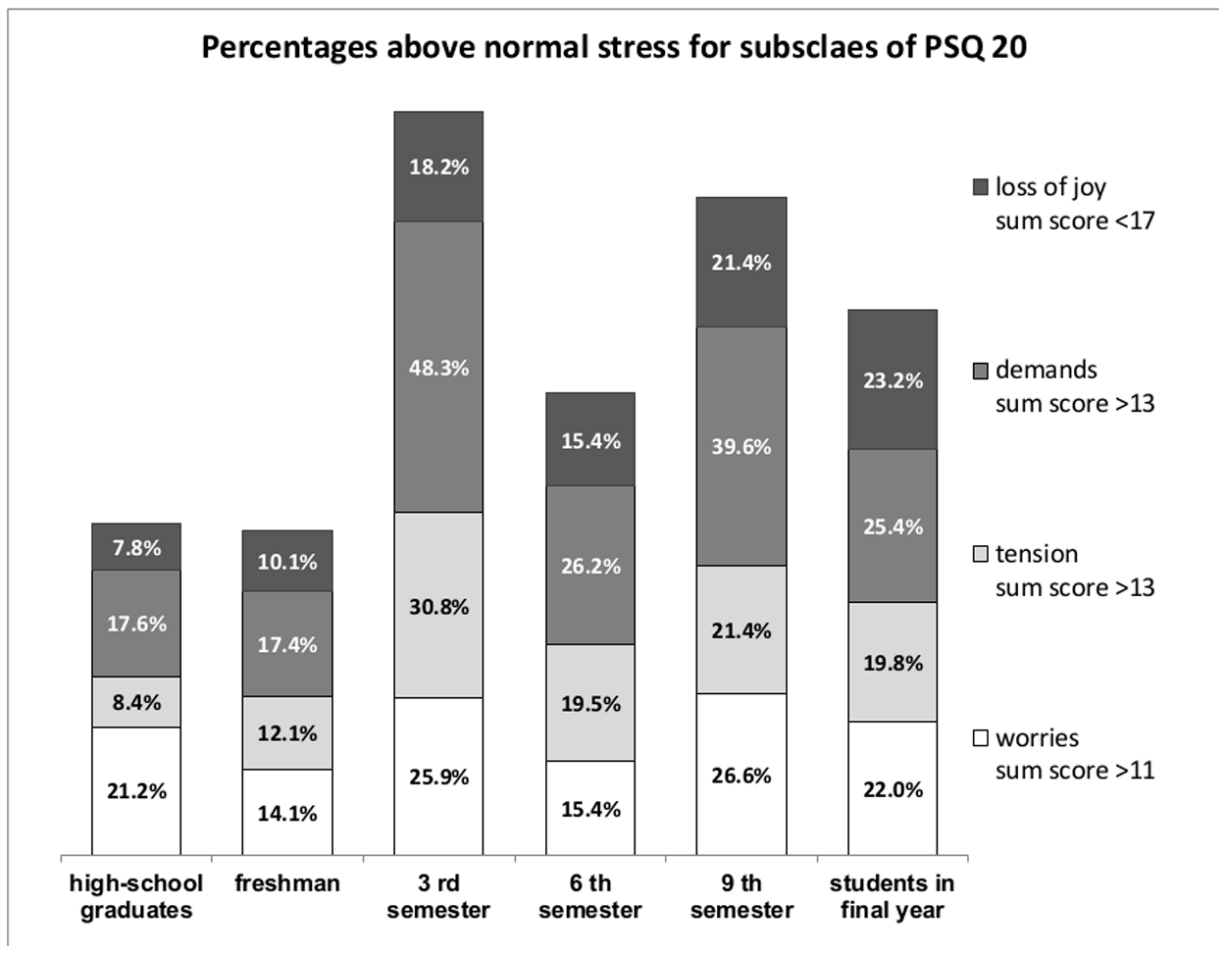
Illustration of the prevalence rates of all subscale values above or below reference sub scores in different stages of medical training. The bars represent the prevalence rate of students and high school graduates in the respective sub-scales of the PSQ-20 above the respective reference values. Significant differences emerged for the sub scales “loss of joy” with (χ^2^ = 32.4; *df* = 5; *p* < .01**). Post hoc analyses yield that students in final year, students in 9^th^ semester and 3^rd^ semester students achieved the highest proportion below the reference values with prevalence rates between 18.2% and 23.2%. 6^th^ semester students had lower rates as these groups with 15.4%, but higher prevalence rates than high-school graduates (7.8%) and freshman students with 10.1%. Significant differences also emerged for the sub scales “high demands” with (χ^2^ = 43.9; *df* = 5; *p* < .01**). Students in 3^rd^ semester and 9^th^ semester had highest proportion above the reference values with prevalence rates of 48.3% and 39.6%. Students in the 6th semester and students in final year had lower rates as these groups with 26.2% and 25.4%. High-school graduates and freshman students achieved rates of 17.6% and 17.4% for “highly demands”. For the sub scale “high tension”, it was also emerged significant differences with (χ^2^ = 67.8; *df* = 5; *p* < .01**). Students in 3^rd^ semester achieved the highest proportion above the reference values with a prevalence rate of 30.8%. Students in the 9^th^ semester, students in final year and students in 6^th^ semester had significantly lower rates as 3^rd^ semester with prevalence rates between 19.4% and 21.4%. On this sub scale high-school graduates and freshman students also achieved lowest prevalence rates above reference value with 8.4% and 12.1%. There was no significant difference for the subscale “high worries” with (χ^2^ = 11.4; *df* = 5; *p* > .05).

For further analyses, the sample was divided into two groups based on cut-off values established by Kocalevent and colleagues [46] and Fliege and colleagues [40] with the same procedure as described above for each of the four PSQ-20 subscales (i.e., demands, worries, tension, and joy). Cut off values for high degree of demands were >13, high degree of worries >11, high degree of tension >13, and low degree of joy <17 (Fig 2).

#### Associations of specific stressors with perceived stress

**Fig 3:**
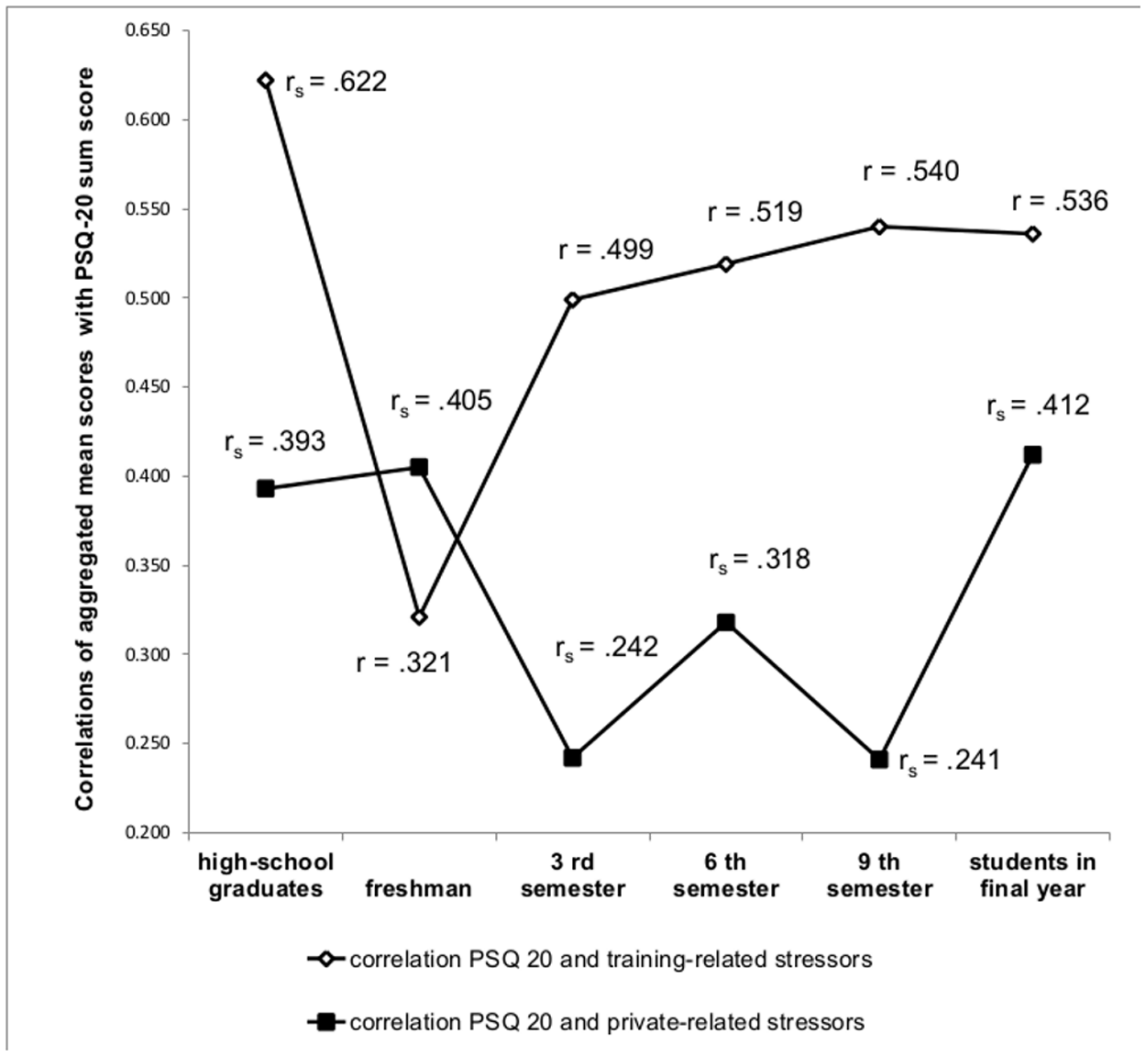
Illustration of the correlations of aggregated mean scores for training-related stressors and private-related stressors with the PSQ-20 total sum score. The two lines symbolize the respective association of the aggregated sum scores for private and training-related stressors with the PSQ-20 sum score. For the aggregation, the single items were summed and a total score was formed. The two lines do not symbolize a progression, but the cross-sectional data points within the investigated subgroups. The correlations were calculated using Spearman rho (r_s_) or Pearson (r) procedures.

With regard to the association of specific stressors and perceived stress (Fig 3), measured with the PSQ-20 total sum score, correlations were higher for the relationship of perceived stress with training-related stressors (r_s_ .303–.624), except in the group of first-year medical students. Private-related stressors were associated more strongly with stress in high-school graduates, freshman students and students in final year than in the 3^rd^, 6^th^ and 9^th^ semester. The correlations between private-related stressors and PSQ-20 total sum scores ranged between .230 and .426.

#### Differential importance of specific private and training-related stressors

Table 3 presents the results of stepwise regression models with single private and training-related stressors for each group of students with one model for high-school graduates, a regression model for freshman students, one model for “medical students” in 3^rd^, 6^th^ and 9^th^ semester and one model for students in final year. The regression model of high-school graduates included only individuals who were certain that they wanted to start medical school, as indicated by a score of ≥9 on a 10-point Likert scale (0 = *uncertain*, 10 = *very certain*) on their certainty of pursuing medical school after graduating from high school. In the following section, results are presented across the different student groups to highlight similarities and differences regarding private- and training-related stressors and demographic control factors. Table 3 also details information about individual single stressors of students in each group.

**Table 3:** Differential relevance of specific private and training-related stressors across subgroups.

**high-school graduates (n = 170)**				model assumptions†
adj. R^2^ = .318				Tolerance > .25
**factors**	**B**	**SE**	**ß**	VIF^♣^ < 4
(intercept)	27.836	1.345		Durbin-Watson: 1.72
missing consultation and support	2.240	.809	.261**	
professional requirements	2.157	.809	.242**	
Study-related-time	1.290	.608	.175*	
conflicts with friends	1.397	.608	.167*	
**freshman medical students (n = 148)**				model assumptions†
adj. R^2^ = .344				Tolerance > .25
**factors**	**B**	**SE**	**ß**	VIF^♣^ < 4
(intercept)	29.553	1.385		Durbin-Watson: 2.01
a-level (others—Germany)	11.061	2.157	.346**	
living situation	1.680	.589	.256**	
contact with other medical students	1.701	.447	.223**	
begin of medical training	1.689	.589	.205*	
**medical students (3**^**rd**^**, 6**^**th**^ **and 9**^**th**^ **semester, n = 412)**				model assumptions†
adj. R^2^ = .312				Tolerance > .25
**factors**	**B**	**SE**	**ß**	VIF^♣^ < 4
(intercept)	28.559	1.071		Durbin-Watson: 1.98
selection and performance pressure	2.073	.392	.241**	
time management & working stiles	1.927	.422	.209**	
timetable	1.927	.421	.293**	
conflicts with parents	1.402	.389	.149*	
gender (female—male)	2.529	.832	.127**	
**students in final year (n = 174)**				model assumptions†
adj. R^2^ = .401				Tolerance > .25
**factors**	**B**	**SE**	**ß**	VIF^♣^ < 4
(intercept)	27.822	1.324		Durbin-Watson: 1.61
time management and work stiles	2.826	.732	.274**	
missing consultation and support	1.510	.613	.170*	
selection and performance pressure	1.618	.733	.166*	
hours to work	1.568	.649	.164*	
conflicts with parents	1.551	.661	.145*	

*p< .05

**p < .01

^**†**^ Model assumptions for linear regression fulfilled

^♣^ Variance Inflation factor

Total variance explanations expressed in adjusted *R*^2^ in the applied models ranged from .312 to .401 for perceived stress as the dependent variable. Model assumptions were fulfilled in all regression models. Except for the group of first-year medical students, the training-related stressors investigated predominantly determined perceived stress at different stages of medical education. Perceived stress in the model of first-year students was predicted by private and demographic control factors (Table 3) only. The most important training-related stressors across groups according to those analyses were s*election and performance pressure* (*β*-values in different groups of .166 to .241) and issues related to *time management and work styles* (*β*-values of .209 to .274). Furthermore, the stressor of *missing consultation and support* was identified as an important training-related stressor, with *β*-values of .170 to .261 among high-school graduates and students in their final year, in the determination of perceived stress.

Referring to private stressors, social conflicts seen as stressors emerged in all regression models. Important stressors included *conflicts with parents* (*β*-values of .145 to .149) for medical students in their third, sixth, and ninth semesters of medical school and students in final year. *Conflicts with friends* were relevant among high-school graduates (*β* = .167), whereas first-year students specified difficult contact with other first-year students as a private stressor (*β* = .223). The private stressor of *living situation* (*β* = .256) was also important among first-year medical students.

Regarding demographic control factors, *gender* made an important contribution to explaining variance among medical students in their third, sixth, and ninth semesters of medical school (*β* = .127). Female medical students between their first and fifth years of study perceived more stress than male medical students. Among first-year students, perceived stress was primarily determined by demographic and private factors, among which “*passed A-level in another country*” was the most important stressor (*β* = .346 explained variance).

The results of the step-by-step regression models for each group of students are shown in Table 3. The regression model for the group of high school graduates only included graduates who were highly confident that they wanted to study medicine (motivation score >9 on the Likert scale from 0 = uncertain to 10 = very certain). The model assumptions are fulfilled for all models.

## Discussion

The current study focused on the relevance of private and training-related stressors associated with perceived stress among students at different stages of medical school and high-school graduates planning to study medicine.

Results confirmed a substantially high burden of perceived stress among medical students at different stages of their medical education with above threshold rates ranging between 10–28%. Medical students in their third and ninth semesters of medical school showed the highest values for perceived stress. To prevent inflated stress rates, students in their fourth and tenth semesters of medical school, who were expected to prepare for major examinations, were excluded. However, two stress rate peaks within the third- and ninth-semester student cohorts indicated that they were possibly affected by the anticipation of major state examinations. Regarding students at early stages of their medical education, high-school graduates and students in the first semester of medical school showed the lowest rates of relevant stress. In line with these results, previous studies have also reported a wave-like trend over time in stress perception of medical students [11–14, 52, 53].

Our study included high-school graduates as a new and important group in order to highlight the earliest possible stage of medical education. Concerning perceived stress, high-school graduates and medical students at the beginning of their medical education had a similar stress pattern, with a similar height of general stress rates and similar stress rates for the PSQ-subscales Joy, Demands, and Tension. The other subgroups of medical students in their third, sixth, and ninth semesters and students in their final year (of practice) during medical training showed a heterogeneous pattern in the Joy, Demands, Worries, and Tension subscales. For the Joy subscale, the pattern across the progress of education follows a linear trend, with lower rates in early stages with high-school graduates and first-year students and the highest rates in later stages, with ninth-semester students and students in their final year. Similar to the two peaks among third- and ninth-semester students for general stress, those two subgroups perceived the highest rates of demands, which clearly indicates high stress. Medical students in their third semester of medical school showed the highest rates of tension, compared to those in early and later stages of medical education. Ultimately, high percentages of worries emerged in all groups of students.

The specific training-related stressors were most strongly associated with perceived stress, except among first-year students. High-school graduates were on the verge of a threshold situation and students in their first semester settled into a position between school and study without any contact with training of medical school. Medical students in their final year find themselves also in a transition phase between finishing medical studies and starting to work as a junior doctor. In these three phases of transitions, private-related stressors showed stronger associations with stress than in the 3^rd^, 6^th^ and 9^th^ semester. In these middle study phases social challenges are less important to perceived stress compared to training related stressors. At the beginning of the medical curriculum training, private related stressors were more strongly associated with perceived stress than training-related stressors.

Current results raise the question of how selection effects before beginning medical school play a significant role in perceived stress in medical students during their medical education. Results in Fig 3 raise the question if high stress burden in medical students is predominantly because of the challenges in medical studies, or if it might be a preexisting trait, i.e. “high-performance motivation”, that becomes evident and maybe virulent during highly demanding medical education. Eppelmann and colleagues [54] demonstrated that stress could prompt behavioural and emotional difficulties and result in an association of stress and emotional and behavioural problems partly mediated by withdrawal from parents. Furthermore, parental pressure could influence medical students’ school performance and result in academic stress [55, 56].

High-school graduates aiming to study medicine were investigated in this study for the first time. Recent results show that high-school graduates in general are already affected by great psychological strain [54]. However, only a third of students of this study reported a high burden of stress characterized by tension, exhaustion, and reduced concentration, whereas only 8.2% of high-school graduates in another study suffered from chronic stress [57].

Four regression models were generated for high-school graduates, freshman medical students, students in their third, sixth, and ninth semesters of medical school, and students in final medical training in order to highlight the heterogeneity of specific stressors that can predict general stress. The most important training-related stressors across the different groups which were endorsed to learning environment were *selection and performance pressure*, *time management & work styles*, and *missing consultation and support*. These stressors reflect students’ subjective needs and fears in the medical curriculum.

Recent qualitative literature [44, 58] supports these stressors as major problem areas endangering wellbeing. Most specifically, Radcliff and Lester [58] qualitatively identified four main stressors among medical students: pressure of work, especially in preparing for examinations; acquiring professional knowledge, skills, and attitudes; transition periods in medical education (school, medical training, preclinical training, clinical training, clinical training for subinterns, and being a physician); and a perceived lack of support from medical school authorities.

Perceived stress in the model of first-year students was predicted by private and demographic control factors only. The most important private-related stressors were social conflicts with parent and friends. The single stressor *conflicts with parents* was highly correlated with *financial problems*, *problems with the living situation* and *jobbing*. It is possible that students deal with different problems such as transitional phases (see Fig 3, explained above) from school to university or from university to practical medical training. Conflicts about financial topics could not be identified as specific stressors, although it is possible that such were hidden within the stressor of conflicts with parents. Other stressors could have been plans for the future and career or different role models.

The demographic control factors of gender and “passing A-level in another country than Germany” showed a relevant contribution to variance respectively, in two subgroups. Among students in their third, sixth, and ninth semesters of medical school, being a woman was a significant demographic stressor. In early and later stages, however, that influence did not reappear. Such results underscore recent results of a higher prevalence of perceived stress among female medical students than among male medical students [7, 59, 60]. Abdulghani and colleagues [61] reported that female medical students perceived more moderate and high stress levels (84.0%) than male (66.5%) medical students. Other studies find the same interaction effect of gender and training stage, in the way that female medical students in their middle stages of medical training show higher stress levels than male students [7, 62–64]. Current literature [1, 16, 23, 65] seeks to explain the gender effect with women being more willing to provide more accurate and honest information about potential psychological disorders than men and to have other behavioural and physiological stress profiles. Hence, the explanation of gender differences is indeed multifactorial, including for example biological and sociocultural dimensions or combinations of the two [66]. With exposure to the medical curriculum, these predispositions and potentially specifically behavioral patterns might lead to increased stress and, with the greater willingness of female students to provide information, to an overall increase in stress rates [1, 17, 60, 64, 66–68].

The stressor “passing A-level in another country than Germany” was the most important stressor at the beginning of medical education, but not at later stages which might be due to a selection effect if the international students with the highest stress do not progress to later stages of medical training.

## Limitations

This study investigated the differential determination of perceived stress according to private and training-related stressors among medical students. The study was conducted in a survey across six subgroups with a total of 1,092 medical students at different stages of medical education and high-school graduates who planned to study medicine. A statistical height of correlations between general stress and specific stressors was identified, and different regression models for each subgroup were required to map the individual influence of private and training-related stressors with perceived stress. This study was conducted as cross-sectional study, and causal inference between perceived stress and specific stressors was not sufficiently confirmed. To further investigate the causal relationships, future longitudinal research with similar cohorts is necessary.

## Conclusion

The results of this study show an alarming burden of stress among medical students at different stages of medical education, with two peaks in the third and ninth semesters. Excluding first-year medical students, training-related stressors were highly associated with perceived stress. Different specific stressors predicted stress at different stages of medical education, which reflects students’ respective fears and needs. Medical students need open, comprehensive information about possible stressors during medical education, private stressors in their daily lives, and possible methods of prevention. Moreover, they should be offered programmes to improve their coping and resilience behaviours and to learn new functional coping strategies.
